# Factors influencing patient falls in a private hospital group in the Cape Metropole of the Western Cape

**DOI:** 10.4102/hsag.v25i0.1392

**Published:** 2020-06-30

**Authors:** Renee Janse van Rensburg, Anita van der Merwe, Talitha Crowley

**Affiliations:** 1 Department of Nursing and Midwifery, Faculty of Medicine and Health Sciences, Stellenbosch University, Stellenbosch, South Africa

**Keywords:** private hospitals, patient falls, intrinsic factors, extrinsic factors, hospitals

## Abstract

**Background:**

The fall rate of patients in hospitals is a worldwide concern due to the impact falls have on patients, the family or relatives, as well as the healthcare setting. Factors influencing patient falls are categorised as intrinsic and extrinsic. Intrinsic factors refers to physical conditions and the extrinsic factors include the environment of the patient, nursing staffing levels and skill mix.

**Aim:**

The study aimed to determine the factors that influence patient falls.

**Setting:**

A private hospital group in the Cape Metropole of the Western Cape.

**Methods:**

A quantitative retrospective descriptive research approach was used by analysing 134 records of patients that have fallen from October 2016 to February 2018. Data was collected using a data extraction sheet and analysed using the Statistical Package for the Social Sciences (SPSS).

**Results:**

Intrinsic factors contributing to patient falls includedthe patient’s age, hypertension, co-morbidities and the use of benzodiazepines as a sedative. Extrinsic factors were the incorrect use of bed rails and the skill mix of the staff. In over half of the cases (*n* = 68; 50.7%), risk assessments were not performed according to the protocol. Only 5 (3.7%) patients sustained major injuries due to the falls. However, the risk of more severe falls increased 2.4 times with the lack of risk assessment.

**Conclusion:**

The lack of accurate and consistent patient fall risk assessments, use of benzodiazepines as a sedative and the staff skill mix were contributors to the fall rate in these hospitals.

## Introduction and background

Patients have a 12% chance of falling during a stay in hospital (Kalisch, Tschannen & Lee 2012:6). A fall is any event that results in a patient being found on the floor. It includes the unplanned or unintentional lowering of a patient to the ground, in the latter case either by visitors or by staff members. Falls are observed or unobserved, often also distinguished as assisted or unassisted falls. An assisted fall occurs in the presence of a staff member who eases or assists the patient to the ground. An unassisted fall occurs when a patient is alone and no one else is present (Staggs, Mion & Shorr 2014:358) to observe the fall or assist the patient.

Factors that most frequently contribute to patient falls are inadequate assessment of patients and communication failures; staff not following procedures and safety measures; deficiency in staff orientation, supervision, leadership, and in the level of the staffing skill mix; and the physical environment surrounding the patient (Sentinel Event Alert 2015:1). Kalisch et al. (2012:6) included the patient’s age and acuity, diagnosis, medication and treatment plan, as well as the layout of a unit where a fall occurs, as factors contributing to patient falls.

According to a study conducted by Bouldin et al. (2013), fall rates in hospitals in the United States range from 3.3 to 11.5 falls per 1000 patient days. The authors differentiate between various hospital units, reporting that fall rates are often higher in neurosurgery, neurology and in medical units in comparison with lower rates in surgical and intensive care units. During their study, they found that the rate of falls with serious injuries was 1.08 per 1000 patient days. They were unable to discern any association between falls and staffing levels or between falls and hospital size (Bouldin et al. 2013).

Groutas and Staggs (2014:40) noted that the international benchmark for patient falls ranges from 2.3 to 7 falls per 1000 patient days. This accounts for approximately 700 000 to 1 000 000 falls per year in the United States. More alarming is the estimate that annually more than 1% (11 000) of these falls are fatal. Unassisted falls inevitably lead to more serious injuries than assisted falls, thus causing greater harm to the patient such as serious fractures or sprains, or even fatal injuries (Groutas & Staggs 2014:40).

Due to the falls benchmarking model (Emergency Care Research Institute [ECRI] 2016) various facilities can be compared with one another using a formula to calculate the fall rate. It is advisable to compare each institution with its own fall history since facilities differ with regard to risk factors such as layouts, patient profiles, and other causative factors (ECRI 2016). Injuries following falls can be categorised as depicted in Table 1.

**TABLE 1:** Categories of injuries due to patient falls.

Category	Description
None	No injury to a patient after the fall
Minor	Application of a dressing, limb elevation, pain relief or attending to bruising
Moderate	Possible suturing, or applying a splint or bandage due to a sprain
Major	Surgery and/or casting due to a fractured limb, skull (including subdural hematomas), ribs or any laceration including a rupture of the liver
Death	Succumbing to the injury following the fall

*Source*: Emergency Care Research Institute (ECRI), 2016, ‘Falls’, *Healthcare Risk Control 14*, 1–49, viewed 15 March 2018, from https://www.ecri.org/components/HRC/Pages/SafSec2.aspx?tab=1.

Despite the use of international best practices and evidence-based procedures, the fall rate with and without serious injuries remains a concern. Various assessment tools are available. These include, for example, the Mors fall scale (Morse, Morse & Tylko 1989), the Johns Hopkins scale (John Hopkins 2007) and the Heinrich II fall risk assessment tool (Hendrich 2007). The hospitals of the private hospital group where this study was conducted made use of the Hendrich II fall risk assessment tool. Despite the use of this tool in the hospitals and the calculation of staffing (including numbers and skill mix) patients continue to fall, resulting in injuries and prolonged hospitalisation. In the context of this study, the categories of nurses in the skill mix include the registered nurse, enrolled nurse, and enrolled nursing auxiliary. A non-nurse category, care worker, also forms part of the skill mix. In general units the percentage registered nurse is 25%, enrolled nurse 35%, and nursing auxiliary 40% per shift.

The reasons for patients’ falls are usually explained in terms of intrinsic or extrinsic risk factors. Intrinsic factors concern a patient’s actual physical condition, while extrinsic factors relate to the environment in which a patient is situated. These include nursing staffing levels and skill mix and are modifiable (ECRI 2016:16).

No studies could be found on the risk factors associated with patient falls in acute hospital settings in South Africa. The aim of this study was to determine the factors that influence patient falls in a private hospital group in the Cape Metropole of the Western Cape.

## Methods

### Study setting

The study was conducted in two private hospitals belonging to the same hospital group in the Cape Metropole, South Africa. Both hospitals are classified as large hospitals with more than 200 beds. Hospital A has 250 acute beds distributed between general surgical, medical, cardio- and neurosurgical, orthopaedic, paediatric, maternity, intensive care, as well as in high care wards. Hospital B has 200 beds located in medical wards, various surgical wards such as vascular, general and gastro-intestinal wards, paediatric and maternity wards, and intensive care units.

### Design

A quantitative research approach utilising a retrospective descriptive study design was selected for this study. Patient documentation, incident reports, copies of electronic capturing events on the hospital data base and appropriate statistical data pertaining to each nursing unit were used as sources of data during this retrospective audit.

### Population and sampling

All the adult falls in all the units in both hospitals over a period of 17 months, namely October 2016 to February 2018, were included in this study. Selection of this time frame was based on the availability of and access to patient records and incident reports in the electronic systems of the two hospitals. A total of 155 falls were recorded in the hospital statistics for this period; 100 falls in Hospital A and 55 falls in Hospital B. At the time of this study, a total of 15 files were not accessible because of filing errors and incomplete nursing notes. Six other folders were excluded since they contained information on falls that occurred in a paediatric and neonatal unit. The final sample consisted of 134 records.

Following a patient fall, an incident report describing the circumstances of the fall is completed by hospital staff. Thereafter, an electronic severity report is compiled to capture the core aspects of the fall. These reports, together with patient records and nursing staff ratio’s, were used as the sources of information for this study.

### Instrumentation

A data extraction sheet was used to retract information retrospectively from patient documentation. The data extraction sheet was a self-designed instrument based on information the researcher obtained from the ECRI study on falls (ECRI 2016), other fall risk assessment tools and the literature.

All aspects, including intrinsic and extrinsic factors, were included in the data extraction sheet. Content and face validity was determined by the supervisor and co-supervisor for the study, as well as by a Nurse Manager and a Nurse Manager General with experience in health services management from the selected hospitals. The instrument was pilot tested with 10 cases prior to the data collection period for the study. Data obtained during the pilot test were not included in the results.

### Data collection

The first author and a trained research assistant extracted data from patient records. Arrangements were made with both the hospitals and the information was made available at the requested times. This included the work space in the hospital, the availability of records as well as the use of electronic data where needed. The researcher and the research assistant gathered the data on the patient falls from the hospitals’ internal electronic database systems, each patient’s hospital folder, and from the hard copies of the incident forms. Completing one of these data collection exercises took approximately 30–43 minutes. Data collection was done on a full-time basis during the first 3 weeks of August 2018.

### Data analysis

Descriptive statistical analysis was performed using the Statistical Package for the Social Sciences (SPSS) with the assistance of a senior biostatistician. Results were displayed using frequency distributions and measures of central tendency and variance. Logistical regression was used to predict whether fall risk assessment predicted the injury severity of a patient measured on a nominal level in this study. A level of significance of alpha < 0.05 were used.

### Ethical consideration

Ethics approval was obtained from the Health Research Ethics Committee (HREC) at Stellenbosch University prior to the commencement of the proposed study (Reference S18/05/097). Thereafter, the proposal was submitted to the Ethics Committee of the private hospital group for further approval and to obtain permission to conduct the study in the two hospitals selected for the study (Reference 251015-048). Due to the retrospective descriptive design of this study, patients’ consent could not be obtained since at the time of the study they had already been discharged. Consequently, a waiver of consent was granted and data was anonymised at the point of data collection and reported in aggregated form.

## Results

### Intrinsic factors

The mean age of the patients who had fallen was 68.37 years (Standard deviation [SD] 15.1). The age range was from 20 to 92 years. More women (*n* = 70; 52.2%) than men (*n* = 64 47.8%) fell during the specified period of data collection in the hospitals involved in this study.

Among the patients implicated in this study, the highest number of falls occurred among general medical patients (*n* = 52; 38.8%), followed by orthopedic patients (*n* = 28; 20.9%), neurovascular/cardiac patients (*n* = 22; 16.4%), surgical patients (*n* = 21; 15.7%) and lastly, mental health patients (*n* = 11; 8.2%).

**FIGURE 1:**
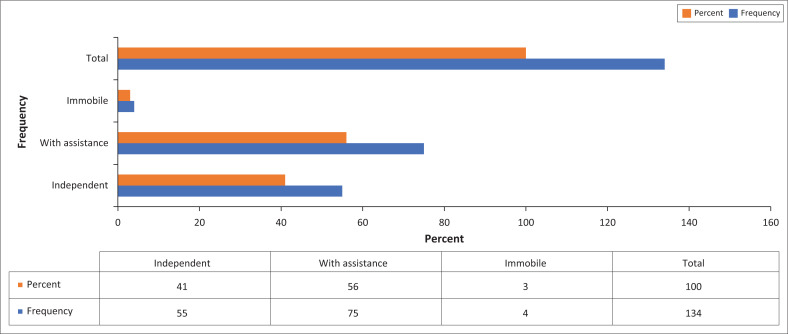
Patient mobility (*n* = 134).

Among the patients who had fallen, 41% (*n* = 55) were independent; 56% (*n* = 75) were mobile with assistance, and 3% (*n* = 4) were immobile patients (Figure 1). It is evident that most of the patients who fell needed assistance with mobility.

With regard to individual health factors, a very small percentage *n* = 6 (4.5%) of the patients were incontinent. Almost a third (32.5%, *n* = 44) of the patients suffered from a mental disorder such as delirium, depression, dementia or confusion. Many (60.4%, *n* = 81) of patients who had fallen suffered from primary or secondary hypertension.

A large percentage (68.7%, *n* = 92) of patients who had fallen had taken benzodiazepines prior to falling compared to anti-epileptics and other CNS suppressants (Table 2).

**TABLE 2:** Medications.

Medication	Response	
	*n*	%
**Benzodiazepines**		
Yes	92	68.7
No	42	31.3
**Anti-epileptics**		
Yes	16	11.9
No	118	88.1
**Other CNS suppressants**		
Yes	48	35.8
No	86	64.2

CNS, central nervous system.

### Extrinsic factors

Furniture and equipment as well as cables and intravenous lines attached to patients were implicated in 13.4% (*n* = 18) of the falls. Wet floors accounted for 16 (11.9%) of the falls (Table 3).

**TABLE 3:** Environment.

Category	Response	
	*n*	%
**Furniture/attachments**		
Yes	18	13.4
No	116	86.6
**Wet floors**		
Yes	16	11.9
No	118	88.1
**Height of toilet**		
Yes	3	2.2
No	131	97.8
**Use of assistance device**		
Yes	7	5.2
No	127	94.8
**Loose shoes/socks**		
Yes	1	0.7
No	133	99.3

A call bell was available to 125 (93.3%) of the patients who had fallen. Five (3.7%) falls occurred in the passage and two (1.5%) in the dining room where no call bell was available.

In most of the reported falls (*n* = 82; 61.2%), the bedrails had not been used. This includes the 11 mental health patients whose beds in the mental unit do not have rails. For this study, 49 (94.2%) of the patients who had fallen from a bed where bed rails were attached, had in fact climbed over the rails.

The majority of the patients fell next to his or her bed (*n* = 74; 55.2%), followed by falls in the toilet or bathroom (*n* = 47; 35.1%); in the patient’s room (*n* = 6; 4.5%), in a passage (*n* = 5; 3.7%), and lastly in the dining room (*n* = 2; 1.5%).

On average the staffing skill mix included one registered nurse, at least two enrolled nurses and enrolled nursing auxiliaries, and one caregiver on duty. The data in Table 4 refer to a maximum of 7 registered nurses on duty per shift which was the case in the intensive care unit.

**TABLE 4:** Nursing skill mix at the time of the falls.

Category	*N*	Minimum	Maximum	Mean	SD
Registered nurse	134	1	7	1.50	1.129
Enrolled nurse	134	0	6	2.38	1.082
Enrolled nurse auxiliary	134	0	4	2.17	0.946
Caregiver	134	0	3	1.01	0.804

SD, Standard deviation.

In most cases (*n* = 131; 97.8%), the staffing at the time of a fall was adequate for the number of patients. The three (2.2%) cases where inadequate staffing was noted, the patients concerned were confused and no staff had been specifically allocated to them.

### Patient falls

The results indicate that most of the falls, 82 (61.2%), occurred at night (Figure 3) and were unassisted (*n* = 131; 97.8%).

**FIGURE 2:**
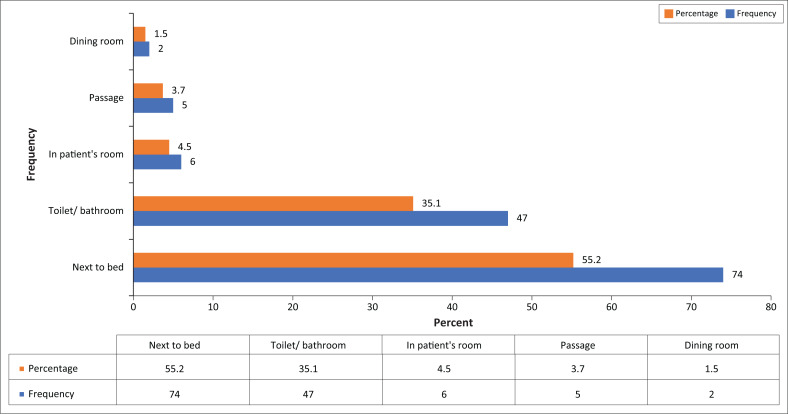
Area of fall (*n* = 134).

**FIGURE 3:**
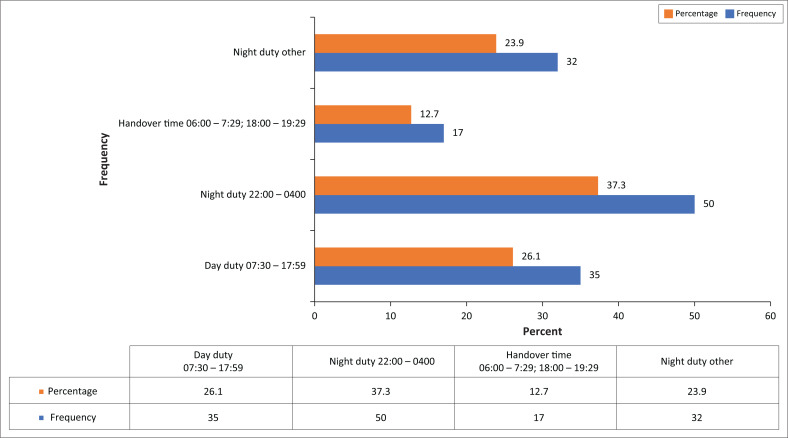
Times of falls (*n* = 134).

In the majority of cases 98 (73.1%) no injuries were sustained, but 24 (17.9%) sustained minor injuries; 7 (5.2%) sustained moderate injuries, and 5 (3.7%) sustained major injuries (refer to Table 5). Only 6 (4.5%) of the patients concerned were subsequently obliged to spend more time in hospital. No deaths due to falls were reported in this study. The injuries sustained during the falls classified as ‘major’ included a fractured head or femur, orbital and humerus fractures, as well as concussion.

**TABLE 5:** Assessment prior to falling.

Variable	Response	
	*n*	%
**Assessment on admission**		
Yes	86	64.2
No	48	35.8
**Assessment prior to fall**		
Yes	85	63.4
No	49	36.6
**Assessment performed on admission and prior to falling**		
Yes	68	50.7
No	66	49.3

### Risk assessment

Only 68 (50.70%) of patients that fell were assessed both on admission and prior to the fall as per the protocol. Four of the five patients who sustained major injuries had not been assessed on admission or before they fell, and thus not following the protocol of the hospital group.

A Hendrich II risk assessment performed according to protocol was significantly correlated with a lower risk of sustaining a more severe injury when compared with the adjacent injury category (*p* = 0.018: odds ratio = 0.42; 95% 0.20–0.86). In other words, the risk of a more severe fall increases 2.4 times if a risk assessment is not performed.

## Discussion

The mean age of all the patients involved in this study was 68.37 years. This age is comparable to the mean age reported in other studies (Hitcho et al. 2004:734; Staggs et al. 2014; Vasilakis et al. 2015:35). Although the Hendrich II risk assessment tool does not include age as a risk factor, other tools such as the Johns Hopkins tool includes the age of 60 and above as a risk factor, with the risk increasing for every 10 years thereafter. In the context of this study, an age above 65 years should be added as a risk factor when nursing assessments are made.

Evident in this study was that more women than men fell. The percentage of women who fell was 52.2%, which corresponds to the data in studies conducted by Hitcho et al. (2004:734), since they also found that women (*n* = 97, 53%) were more inclined to fall. Rafferty et al. (2007) also noted that more women (51.9%) fell when compared to men. Similarly, Vasilakis et al. (2015:35) reported that 54% of the patients who fell were women. In contrast, however, Staggs et al. (2014) found that men had a higher risk of falling when compared to women since men are more reluctant to request assistance with mobilisation than women. In this study, a particular gender was not identified as being at risk of falling. However, this does not correspond to the recommendations that Hendrich (2007) and her colleagues incorporated in the Hendrich II fall risk assessment form. They recommended that because of their finding that men generally have a higher risk of falling, all men receive a score of one. This could potentially lead to women being overlooked or wrongly classified as having a low risk of falling. Neither the Morse fall risk assessment tool nor the Johns Hopkins tool assesses gender as a factor in falling.

From the research data it was evident that most patients (80.6%) had one or more co-morbid condition. Watson, Salmoni and Zecevic (2015:88) noted that patients with multiple diseases had an increased risk of falling and concluded that the risk of falling increases in patients with a primary medical condition and more than one secondary condition. The Hendrich II tool does not include co-morbidities as a fall risk factor. The Morse risk assessment tool makes provision for the inclusion of a secondary diagnosis as part of assessment, which increases the fall risk by 25 points and places a patient in a low risk category requiring standard fall prevention measures. In the context of this study, it might be advisable to include a secondary diagnosis as a risk factor.

In this study, 60.4% or 81 patients had hypertension. Watson et al. (2015:89) stated that patients who take anti-hypertensive medication have an increased risk of falling. In their study, Hitcho et al. (2004:734) noted that 34.4% of the patients who had received anti-hypertensive medications had fallen in hospital. None of the fall risk assessment tools discussed in this study, assessed blood pressure-related risk among patients. The Johns Hopkins tool identifies the use of anti-hypertensive medication as a risk factor. In this study, with the high percentage of patients that had hypertension, this should be added as a risk factor to alert the staff of the risk of falling.

Sensory disturbances did contribute to patient falls in this study as 32.5% (*n* = 44) of the patients suffered from confusion, delirium, depression and dementia. Other studies have found that 36% – 44% of patients who fell had a diagnosis of delirium or a mental disorder (Hitcho et al. 2004:734; Vasilakis et al. 2015:40). The Hendrich II tool assesses for confusion, disorientation and impulsivity of patients, while the Morse tool assesses whether a patient is aware of his or her ability and limitations. The Johns Hopkins tool assesses a patient’s cognition.

Medication administered to patients prior to the fall was identified as a contributing factor in patient falls. A high number of patients that fell (68.7%) had received a dose of benzodiazepines within an 8-h period before they fell. In this study, it was found that Stillnox (zolpidem tartrate) was also predominantly administered to patients. Stilnox is indicated for the treatment of insomnia. Its effects are similar to that of benzodiazepines, but its structure and molecular components are different. Stillnox interacts with the GABA-benzodiazepine receptor complex and shares some pharmacologic properties with benzodiazepines (Foda & Ali 2010).

Environmental factors contributed to the falls of 18 (13.4%) patients. This includes falls involving ward furniture or equipment, for example, patients tripping over the cords of equipment. Floors that were wet, either because the patient had showered or urinated on the floor, played a role in 11.9% of falls. Similarly, Hitcho et al. (2004:736) reported that wet floors did not play a significant part in falls, and only 6% of the falls relevant to their study were related to either water or urine on the floors. This is a slightly lower percentage than the aforementioned percentage for this study.

A call bell was available for all but three patients prior to the fall. Hitcho et al. (2004:736) note that only in 3% of the cases implicated in their study had the call bell been used prior to the fall. Approximately 24% of these patients said they thought they did not need assistance to become mobile and therefore did not call for assistance. This corresponds with a finding of the present study, namely that, according to the incident reports written by the nursing staff, patients had stated that they did not want to disturb the nurses and felt they were able to become mobile on their own.

In this study, five of the 12 moderate and major injuries occurred where patients climbed over the bed rails, including three of the five major injuries sustained. In order to assess the value of bedrails, a study needs to be conducted with a comparison group of patients that did use the bed rails and did not fall. Hignett and Masud (2006:608) reported that there was no clear evidence that raised bed rails prevented patients from falling, and instead suggested that the bed should be lowered to a height where a patient could actually touch the ground while lying on the bed. This would also reduce the severity of injuries should a patient fall (Hignett & Masud 2006:609).

More falls occurred in the medical units followed by the surgical units. This corresponds with information in the literature, notably in the report by Watson et al. (2015) who stated that many of the falls occurred in the medical units, followed by falls in surgery, and then by falls in the neurological and cardiac units. Similarly, Vasilakis et al. (2015:35) reported that medical patients contributed to 57.3% of the falls in their study, surgical patients to 22.3%, and patients in other categories to 20.4%. In a study conducted in Norway by Lerdal et al. (2018:1826), it was found that the majority of falls occurred in the medical wards.

The areas where the majority of falls occurred was 55.2% next to the bed, followed by 35.1% in the bathroom. Hitcho et al. (2004) reported that a high percentage of falls (*n* = 155; 84.7%) occurred in the patients’ rooms, while 20 (10.9%) happened in the bathroom and eight (4.4%) in other areas. Hignett et al. (2010) found in their study that the majority of falls happened at the bed of the patient and the second highest area of falls was in the bathroom. This corresponds with the results of the present study.

Hitcho et al. (2004:735) reported that most falls (*n* = 107; 58.5%) occurred during the 19h00–06h59 time frame. This corresponds with a finding of the present study. Watson et al. (2015:89) found that the highest number of patients fell during the 10h00–12h00 time frame, a situation they ascribe to various investigations that take place in that period, thereby reducing the nursing hours. They also commented that more falls were reported during the night–duty time frame of 01h00–02h00, which may have been due to the smaller number of nursing staff on duty at night (Watson et al. 2015:89).

Although the number of staff at the hospitals met the specifications of the company staffing model, the skill mix is questionable. The median registered nurse in this study per shift was one, although certain surgical and orthopaedic units reportedly had two Registered Nurses (RN) on duty per shift during the day. This is concerning since some of the units had up to 44 beds with an average occupancy of 64.3%. According to Aiken et al. (2016) the rate of adverse events happening among patients drops significantly with an increase in registered nurses on duty. However, in their study, Bouldin et al. (2013) noted no trend between patient falls and the staffing level.

Most of the falls relevant to this study were unassisted (97.8%). This corresponds with information in published reports which stated that more unassisted than assisted falls took place, for instance Staggs et al. (2014:358) noted that 85.5% of the falls reported in their study were unassisted, while Hitcho et al. (2004:735) commented that in their study they found that 145 (79.2%) were unassisted falls and 15 (8.2%) were assisted falls. The percentage of unassisted falls is, therefore, higher in this study when compared with falls reported in the other studies.

The findings of this study relating to the assessments done in the hospitals are perturbing. Only 68 (50.7%) patient assessments were conducted as per protocol, namely on admission and at each shift change. In their study, Dykes et al. (2009) stated that the fall assessment tool is both necessary and important in fall prevention. However, this becomes insignificant if the findings of an assessment are not communicated to all staff, and no individual care plan is devised for a patient at risk. In their study of falls in the Emergency department, Berry et al. (2018:39) found that of the 53 patients that fell, 39 or 73.6% had a fall risk assessment completed which is similar to the 64.2% of patients who received a fall risk assessment on admission in the present study.

## Recommendations

Recommendations include the revision of risk assessment tools to incorporate context-specific factors, adherence to procedures regarding risk assessments as well as auditing the result of these assessments. Attention should be given to current skill mix ratios; an increase in the registered nurse category is proposed to align with international norms.

## Limitations

A limitation of the study was the small sample size since only two hospitals from the same hospital group were involved in the project. There was no comparison group with which findings could be compared. Incomplete nursing notes in patient folders as well as the fact that nursing staff had not completed the documents accurately made it difficult to extract data. The risk assessment document as well as the treatment chart in many of the patient folders had not been adequately completed. To compensate for this shortcoming, the information required to complete the data extraction form was obtained from incident reports and electronic documents created by the hospitals’ quality assurance department after the falls had occurred.

## Conclusion

This study provided information on the number of patients who fell over a 17-month period in two specific hospitals in the Western Cape. The intrinsic factors that were identified in the study as contributing to patient falls were identified as age, hypertension, co-morbidities, and the use of benzodiazepines as a sedative. The extrinsic factors included the skill mix of the staff and inappropriate use of bed rails. Patient falls in hospitals remains a patient safety concern. Further studies are recommended to address the factors involved and to substantially reduce the number of patient falls in hospitals.
