# Erratum: Factors influencing patient falls in a private hospital group in the Cape Metropole of the Western Cape

**DOI:** 10.4102/hsag.v26i0.1773

**Published:** 2021-12-01

**Authors:** Renee Janse van Rensburg, Anita van der Merwe, Talitha Crowley

**Affiliations:** 1 Department of Nursing and Midwifery, Faculty of Medicine and Health Sciences, Stellenbosch University, Stellenbosch, South Africa

In the version of this article initially published, Janse van Rensburg, R., Van der Merwe, A. & Crowley, T., 2020, ‘Factors influencing patient falls in a private hospital group in the Cape Metropole of the Western Cape’, *Health SA Gesondheid* 25(0), a1392. https://doi.org/10.4102/hsag.v25i0.1392, the article section was given incorrectly. The correct section should be Original Research instead of Review Article.

This correction does not alter the study’s findings of significance or overall interpretation of the study’s results. The publisher apologises for any inconvenience caused.

